# Isolation, characterization, and immunomodulatory activity evaluation of probiotic strains from colostrum and canine milk

**DOI:** 10.3389/fvets.2023.1266064

**Published:** 2023-11-23

**Authors:** Sandra Rayén Quilodrán-Vega, Carolina Muñoz-Flores, Ana Pino, Paula Buldres, Felipe Sandoval, Alex Aguirre, Brígida Portillo, Natalie Parra, Claudia Altamirano, Leonardo Albarracín, Julio Villena, Jorge R. Toledo

**Affiliations:** ^1^Laboratory of Food Microbiology, Faculty of Veterinary Sciences, Universidad de Concepción, Chillán, Chile; ^2^Biotechnology and Biopharmaceuticals Laboratory, Departamento de Fisiopatología, Facultad de Ciencias Biológicas, Universidad de Concepción, Concepción, Chile; ^3^Laboratorio de Cultivos Celulares, Escuela de Ingeniería Bioquímica, Pontificia Universidad Católica de Valparaíso, Valparaíso, Chile; ^4^Laboratory of Immunobiotechnology, Reference Centre for Lactobacilli (CERELA-CONICET), San Miguel de Tucumán, Argentina

**Keywords:** probiotic, gastrointestinal infection, dog, immunomodulatory lactic acid bacteria, milk strains

## Abstract

**Background:**

This study aimed to characterize potential probiotic strains for use in dogs to prevent infectious enteropathies. Lactic acid bacteria (LAB) isolated from canine milk and colostrum were characterized according to their functional properties, including their resistance to gastrointestinal conditions, inhibitory effect against pathogens, and intestinal adhesion.

**Methods:**

The immunomodulatory effects of the strains were also analyzed in *in vitro* and *in vivo* studies. Among the strains evaluated, two LAB strains (TUCO-16 and TUCO-17) showed remarkable resistance to pH 3.0, bile salts, and pancreatin, as well as inhibitory effects against pathogenic *Escherichia coli, Salmonella* sp., and *Clostridium perfringens*.

**Results:**

The TUCO-16 and TUCO-17 strains induced a significant increase in the expression of TNF-α, IL-8, and TLR2 in canine macrophages. The oral administration of TUCO-16 and TUCO-17 strains to mice significantly augmented their resistance to pathogenic *E. coli* or Salmonella intestinal infections. Both canine strains reduced intestinal damage and pathogen counts in the liver and spleen and avoided their dissemination into the bloodstream. These protective effects were related to the ability of TUCO-16 and TUCO-17 strains to differentially modulate the production of IFN-γ, IFN-β, TNF-α, IL-6, KC, MCP-1, and IL-10 in the intestinal mucosa.

**Conclusion:**

Both strains, TUCO-16 and TUCO-17, are potential probiotic candidates for improving intestinal health in dogs, particularly for their ability to inhibit the growth of Gram-negative pathogens common in gastrointestinal infections and modulate the animal's immune response. Further studies are required to effectively demonstrate the beneficial effects of TUCO-16 and TUCO-17 strains in dogs.

## 1 Introduction

In the last decade, great advances have been made in the knowledge of the composition of the intestinal microbiota of animals like dogs. Most microorganisms detected in the canine gastrointestinal tract belong to the *Bacillota* (*Firmicutes*), *Fusobacteriota* (*Fusobacteria*), *Bacteroidota* (*Bacteroidetes*), *Pseudomonadota* (*Proteobacteria*), and *Actinomycetota* (*Actinobacteria*) phyla (1). A healthy dog's microbiome is stable; however, its health can become destabilized due to age, diet, and other environmental factors, leading to dysbiosis; for example, acute and chronic intestinal inflammation, where intestinal disturbances result in functional changes in the microbial transcriptome, proteome, or metabolome (2, 3). The microbiomes of dogs and humans are structurally and functionally similar, implying that human studies are predictive in dogs and vice-versa, obtaining a double benefit in research (4).

Dogs, like humans, can develop infectious enteropathies, characterized by a duration of up to 3 weeks with symptoms such as diarrhea, vomiting, nausea, abdominal pain, and weight loss, among others. Enteropathies can be reversed in dogs with a change in diet, administration of antibiotics, or immunosuppressants (5). Among dogs' most used antibiotics for treating enteropathies are metronidazole, amoxicillin, tylosin, and lincomycin (6–8). The use of antibiotics can improve the clinical symptoms of patients with gastrointestinal infections; however, they cause negative alterations in the intestinal microbiota, and there is also concern about the selection of bacteria resistant to antibiotics (9). Studies have reported that the number of multiresistant isolates is increasing and can be transmitted between animal bacteria and the human microbiota, which is a big problem for public health (10–12). Additionally, antibiotic-associated gastrointestinal signs, such as marked hyporexia, vomiting, and diarrhea, may lead to premature antibiotic treatment discontinuation (13). Thus, alternative preventive and therapeutic alternatives are urgently needed to significantly reduce these disorders in dogs.

Probiotic microorganisms have been proposed as alternatives to antibiotics to improve protection of the intestinal mucosa (14, 15). Human probiotics are an alternative therapy for health maintenance in pets; however, the most appropriate source of probiotics should be derived from the pet itself (16). The control of intestinal pathogens in pets is a growing concern and it is desirable to select native probiotic strains that exhibit host specificity to cope with intestinal conditions associated with domestication and multiresistant pathogenic bacteria (17–19). Isolation from the host has been a valuable source for identifying strains with beneficial probiotic characteristics for the intestinal health of pets, avoiding the use of human-derived probiotics (20, 21). In these studies, strains were identified that improve fecal parameters in dogs with diarrhea, their nutritional status, a decrease in coliform counts, an increase in lactic acid bacteria (LAB), and an increase in hemoglobin and serum magnesium. In addition, the administration of these probiotic strains improves the food intake, the weight of the animals, and their immunity and modulates the intestinal microbiota in dogs of different ages (22). Despite the promising results obtained with LAB strains isolated from dogs, there are no studies demonstrating their efficacy to beneficially modulate immunity against pathogens.

In the present work, we analyzed the properties of a bank of strains isolated from canine milk and colostrum in terms of their resistance to gastrointestinal conditions, inhibitory effect on pathogens, intestinal adhesion as wells as immunomodulatory effects in *in vitro* and *in vivo* studies, with the aim of selecting strains that could be used for designing a new canine probiotic formulation to prevent gastrointestinal disorders in dogs.

## 2 Materials and methods

### 2.1 Sample collection

Canine milk and colostrum were extracted aseptically from female dogs by a veterinary professional. Maternal milk and colostrum samples were collected aseptically by washing the nipple area with a soap and water solution to avoid contamination with other microorganisms from the skin. The veterinary wear appropriate sterilized gloves during the procedures. After collection, the samples were transferred to the laboratory of Food Microbiology, of the Faculty of Veterinary Sciences from the Universidad de Concepción (Chillán city, Chile). The samples were inoculated in Man Rogosa and Sharpe (MRS) broth and incubated in microaerophilia, at 37°C, for 48 h. The bacteria were then streaked on MRS agar and the plates were incubated under the mentioned conditions. As control strains, the probiotic *Lacticaseibacillus casei* Shirota and the strain *Lactobacillus acidophilus* of the commercial probiotic BIOPOWER (CPB) were used. In addition, an immunomodulatory strain from pig's breast milk (TUCO-4) belonging to the lactobacilli group was also used (23).

### 2.2 Strains characterization

Canine LAB strains were studied based on their macroscopic and microscopic characteristics according to Gram stain, colony morphology, and catalase test. Their functional characteristics were also determined according to their resistance to bile salts (Oxgall 0.5 and 5.0% w/v), NaCl (2.0, 6.0 and 9.0% w/v), pancreatin (0.5, 1.0 and 2.0% w/v) and to acid (pH 3), as described previously (23, 24). Briefly, the strains were cultured in MRS broth with bile salts, NaCl, pancreatin and at pH 3 (adjusted with HCl 1M) and incubated in microaerophilia, at 37°C for 24 h. Viability was tested on MRS agar and incubated in the same mentioned conditions.

### 2.3 Antimicrobial activity

The effect of canine LAB strains against pathogens was analyzed in soft agar or semi solid agar (24). The bacterial pathogens used in these experiments were *E. coli* ATCC 25922, an *E. coli* enterotoxigenic strain (ETEC), *Salmonella enterica* ATCC 13076, a clinical isolate of *Salmonella* sp., and *Clostridium perfringens* NCTC 13170. LAB strains were washed twice with Butterfield's Buffer (5,000 rpm for 5 min), and adjusted to 0.5 McFarland. Drops were spotted on the surface of MRS agar and were allowed to dry for 45 min. The plates were incubated in microaerophilia, at 37°C for 48 h. Each pathogen strain was adjusted to 0.5 McFarland and 1 mL was added to 9 mL of soft agar (75%) or thioglycolate medium (for *C. perfringens*). Plates were incubated aerobically at 37°C for 24 h and anaerobically for *C. perfringens* cultures. The diameters of the inhibition zones were measured.

### 2.4 Presumptive safety profile

Canine LAB strains were tested according to presumptive safety, on sheep blood agar and in gelatin medium (23). The strains were streaked on sheep blood agar and incubated at 37°C for 48 h in microaerophilic condition. For gelatinase detection, the strains were streaked deep in gelatin medium and incubated in the same conditions mentioned above. After this period, the tubes with the cultures in gelatin medium were placed at 4°C for 2 h to verify liquefaction action. In addition, canine LAB strains were analyzed for their antibiotic resistance profile toward antibiotics discs (amikacin 30 μg, erythromycin 15 μg, vancomycin 30 μg, ciprofloxacin 5 μg, gentamicin 20 μg, amoxicillin 25 μg, tetracycline 30 μg and ampicillin 10 μg) that were placed on MRS agar surface with the tested LAB strain adjusted to 0.5 McFarland concentration. The plates were incubated at 37°C for 48 h in microaerophilia. The diameters of the inhibition zones were measured.

### 2.5 Classification of the strains

The selected canine strains (TUCO-16 and TUCO-17) were classified by conventional PCR to determine their membership in the actual lactobacilli group using the LbG primers (forward 5′AGAAGAGGACAGTGGAAC and reverse 5′TTACAAACTCTCATGGTGTG). The DNA was extracted according to the instructions of the supplier of the Mo bio commercial kit (Carlsbad, CA USA). *L. casei* Shirota was used as positive control and the strain *Staphylococcus aureus* ATCC 29213 as negative control (25).

### 2.6 Immunomodulatory activity *in vitro*

The immunomodulatory activity of the canine LAB strains was evaluated in the canine macrophage cell line DH82. The porcine strain TUCO-4 and the probiotic CPB were used for comparison. DH82 cells were seeded in 24-well plates until 80% confluence. Canine macrophages were stimulated for 12 h with the different bacterial suspensions (10^6^, 10^7^, 10^8^ or 10^9^ CFU/mL) of TUCO-16, TUCO-17, TUCO-4 or CPB strains. Negative controls without treatment for 12 and 24 h were included. All conditions were performed in triplicate. Bacterial resuspensions were prepared with DMEM culture medium supplemented with 10 % fetal bovine serum (FBS), 2 mM L-glutamine, and non-essential amino acid (NEAA) solution. Cells were grown at 37°C with 5% CO_2_. After the stimulations, RNA extraction was performed using the Trizol reagent (Invitrogen, Thermo Fisher Scientific, Waltham, MA, USA), according to the manufacturer's instructions. RNA samples were quantified with the Synergy HTX microplate reader (BioTek Instruments, USA), integrity was verified by agarose gel electrophoresis and the samples were stored at −80°C. Real-time PCR (RT-PCR) assays were performed to assess the relative expression of the pro-inflammatory cytokines *TNF-*α and *IL-8*, and the pattern recognition receptors (PRRs) *TLR2* and *NOD2*. *GAPDH* expression was used as a normalizing gene (Table 1). For the synthesis of complementary DNA and real-time PCR, the Brilliant II SYBR^®^ Green QRT-PCR Master Mix, 1-Step commercial kit (Agilent, USA) was used in the AriaMx Real-Time PCR System (Agilent, USA). The results were analyzed using the comparative method of Ct (2^−ΔΔCt^).

**Table 1:** Sequences of DNA oligos used in RT-PCR for the canine cell line.

**Gene name**		**Sequence 5^′^-3^′^**	**Amplicon size (bp)**	**GenBank accession number**
*TNF- α*	Forward	TAGCAAACCCCGAAGCTGAG	118	NM_001003244.4
	Reverse	TACAACCCATCTGACGGCAC		
*IL-8*	Forward	TGTCCTTTCTGCAGCTCTCTG	138	NM_001003200.1
	Reverse	GGGCCACTGTCAATCACTCT		
*TLR2*	Forward	GGACGTCTGTTATGACGCCT	135	NM_001005264.3
	Reverse	CCGGGAATAAAGTCCCGCTT		
*NOD2*	Forward	TTGGCTGCCTTCCTTCTACG	135	NM_001287039.1
	Reverse	GGTGCTCAGAAAGCGAGACT		
*GAPDH*	Forward	GTCCCCACCCCCAATGTATC	98	NM_001003142.2
	Reverse	TCCGATGCCTGCTTCACTAC		

### 2.7 Immunomodulatory activity *in vivo*

Female 5-week-old BALB/c mice were obtained from the closed colony kept at CERELA-CONICET (Tucumán, Argentina). Animals were housed in plastic cages in a controlled room (22 ± 2°C temperature, 55 ± 2% humidity) with a 12 h light/dark cycle. Mice were housed in plastic cages and environmental conditions were kept constant, in agreement with the standards for animal housing. Animal welfare was in charge of researchers and special staff trained in animal care and handling at CERELA. The minimal number of mice required for an appropriate statistical analysis was calculated with the help of the Biostatistics Laboratory of CERELA. Mice health and behavior were monitored twice a day. Animals were euthanized immediately after the time point was reached by using xylazine and ketamine. No signs of discomfort or pain were observed before mice reached the endpoints. No deaths were observed before mice reached the endpoints.

All experiments were carried out in compliance with the Guide for Care and Use of Laboratory Animals and approved by the Ethical Committee of Animal Care at CERELA, Argentina (protocol numbers BIOT-CRL/14 and BIOT-CRL/11).

The strains TUCO-16 and TUCO-17 were orally administered to different groups of mice for five consecutive days at a dose of 10^8^ cells/mouse/day (Supplementary Figure 1). The LAB-treated groups and the untreated control mice were fed a conventional balanced diet *ad libitum*. One day after the last LAB administration (day 6) animals were challenged with pathogenic *E. coli* or *Salmonella*.

In the first set of experiments, animals were orally infected with a mouse adapted enterotoxigenic *E. coli* (ETEC) K88 strain (1 × 10^9^ cells) diluted with 0.1 M carbonate buffer (pH 9.0) (26). Two days after the infection, the mice were sacrificed to collect the jejunum, ileum, spleen, and liver samples. The collected tissues were weighed and homogenized in BHI broth. Homogenates were plated on the kanamycin resistant MAC agar medium for ETEC counts. Results were expressed as log of colony-forming units (CFU) per gram of organ. Serum biochemical markers of injury as well as intestinal cytokines concentrations were also evaluated 2 days after ETEC challenge as described below.

In the second set of experiments, treated and control mice were challenged with 50 μl of 10^7^ cells/mouse of *Salmonella typhimurium* (20LD50) by oral administration (27). An aliquot (200 μL) of the intestinal pathogen from an overnight culture was placed in 5 mL of sterile BHI broth and incubated for 4 more hours (37°C, aerobiosis). The concentration of *Salmonella* was adjusted to 1 × 10^7^ CFU in PBS. Two days after the infection, the mice were sacrificed to collect the jejunum, ileum, spleen, and liver samples. The collected tissues were weighed and homogenized in BHI broth. Serum biochemical markers of injury as well as intestinal cytokines concentrations were also evaluated 2 days after *Salmonella* challenge as described below.

Lactate dehydrogenase (LDH) and aspartate aminotransferase (AST) activities were determined in the serum to evaluate gastrointestinal injury indirectly. Blood samples were obtained through cardiac puncture under anesthesia. LDH and AST activities, expressed as units per liter of serum, were determined by measuring the formation of the reduced form of nicotinamide adenine dinucleotide (NAD) using the Wiener reagents and procedures (Wiener Lab, Buenos Aires, Argentina) (26).

Intestinal fluid samples were obtained as described before (26). Briefly, the small intestine was flushed with 5 mL of PBS and the fluid was centrifuged (10,000 g, 4°C 10 min) to separate particulate material. The intestinal supernatant samples were kept frozen at −80°C until use. Tumor necrosis factor (TNF)-α, IL-6, IL-10, IL-15, interferon (IFN)-β and IFN-γ, chemokine KC (or CXCL1), and MCP-1 concentrations in intestinal fluid a were measured with commercially available enzyme-linked immunosorbent assay (ELISA) technique kits following the manufacturer's recommendations (R&D Systems, MN, USA).

### 2.8 Statistical analysis

The following programs for statistical data analysis were combined and used: software GraphPad Prism 8 (GraphPad Software Inc., San Diego, CA, USA) and Excel (Microsoft 365). Statistical significance of the results was evaluated using one-way ANOVA and Dunnett's multiple comparison test. The level of statistical significance was defined as *p* < 0.05.

## 3 Results

### 3.1 Selection of potential probiotic canine LAB strains

Five strains from canine breast milk (TUCO-17, TUCO-2P1, TUCO-2P2, TUCO-3P1, and TUCO-3P2), and one strain from canine colostrum (TUCO-16) were isolated and characterized for their functional properties (Table 2). Probiotic strains TUCO-4, Shirota and CPB were used for comparisons. All the isolated strains were Gram positive, of which only two strains presented bacillary morphology: TUCO-16, and TUCO-17. The functional characteristics of the strains showed that all of them were resistant to pH 3 at 24 h (Table 2). The TUCO-16 and TUCO-17 strains stand out, presenting resistance between 0.5 and 5.0% w/v of bile salts, resistance in the range of 2.0 to 9.0% w/v of NaCl, and were resistant at all concentrations of pancreatin used in this study, similar to the control strains (Table 2). Thus, the results showed that these two canine strains could resist gastric conditions. The strains TUCO-2P1 and TUCO-3P2 were resistant at 5% w/v of bile salts and in the rage of 2.0–9.0% w/v of NaCl, while the strain TUCO-2P2 did not growth in the presence of bile salts neither pancreatin concentrations but did grow at 2% w/v of NaCl. The strains TUCO-2P1 and TUCO-3P1 were resistant to pancreatin in the rage of 0.5–1.0% w/v and the strain TUCO-3P2 did not resist the pancreatin concentrations (Table 2).

**Table 2:** Functional characteristics of strains isolated from canine breast milk and colostrum.

**LAB strains**	**Morphology**	**Bile salts (% p/v)**	**NaCl (% p/v)**	**Pancreatin (% p/v)**	**pH 3**
TUCO-2P1	Cocci	5.0	2.0–9.0	0.5–1.0	+
TUCO-2P2	Cocci	No growth	2.0	No growth	+
TUCO-3P1	Cocci	No growth	No growth	0.5–1.0	+
TUCO-3P2	Cocci	5.0	2.0–9.0	No growth	+
TUCO-16	Rod	0.5–5.0	2.0–9.0	0.5–2.0	+
TUCO-17	Rod	0.5–5.0	2.0–9.0	0.5–2.0	+
TUCO-4	Rod	0.5–5.0	2.0–9.0	0.5–2.0	+
Shirota	Rod	No growth	2.0–9.0	0.5–2.0	+
CPB	Rod	0.5–5.0	2.0–9.0	0.5–2.0	+

The ability of canine LAB to inhibit the growth of intestinal pathogens was analyzed (Table 3). All the studied strains, including TUCO-16 and TUCO-17, showed inhibitory effect against *C. perfringens*, with inhibition halos > 30 mm of diameter. The TUCO-16 and TUCO-17 strains showed also inhibitory effect against the pathogenic *E. coli* and *Salmonella* strains (Table 3), and they were as effective as the Shirota and CBP strains. However, the canine strains were less efficient than the porcine TUCO-4 strain to limit the growth of pathogenic *E. coli* and *Salmonella* (Table 3). The strains TUCO-2P1, TUCO-2P2, TUCO-3P1, and TUCO-3P2 were also evaluated in their abilities to inhibit intestinal pathogens but no inhibition zones were found (data not shown). Considering these results, the strains TUCO-16 and TUCO-17 were selected for further experiments. The TUCO-16 and TUCO-17 strains were identified to belong to the actual lactobacilli group by conventional PCR.

**Table 3:** Inhibition of intestinal pathogens by of strains isolated from canine breast milk and colostrum.

**Strains**	***E. coli***	***Salmonella enterica***	***Salmonella* sp**.	**Enterotoxigenic *E. coli***	***C. perfringens***
TUCO-16	27.7 ± 2.0_ab_	26.3 ± 0.8_b_	24.3 ± 2.0_ab_	27.7 ± 2.7_c_	47.0 ± 2.4_bc_
TUCO-17	31.7 ± 2.5_c_	25.5 ± 2.3_b_	25.3 ± 1.6_b_	25.5 ± 0.8_b_	46.2 ± 2.4_b_
TUCO-4	48.5 ± 1.5_d_	38.3 ± 1.9_c_	38.7 ± 1.9_c_	34.5 ± 2.1_d_	36.0 ± 1.3_a_
Shirota	26.3 ± 0.5_a_	21.3 ± 0.8_a_	23.2 ± 0.8_a_	20.5 ± 0.8_a_	48.8 ± 1.0_c_
CPB	28.5 ± 1.4_b_	24.8 ± 1.3_b_	24.8 ± 0.8_ab_	26.3 ± 0.8_bc_	46.7 ± 1.0_bc_

Halos are shown in diameters (mm).

Different letters indicate significant differences (*p* < 0.05) in the columns. ± sign indicates standard deviation.

### 3.2 Presumptive safety profile

The presumptive safety characteristics of the selected strains are summarized in Table 4. TUCO-16 and TUCO-17 strains did not show hemolytic or gelatinase activity. The canine strains presented antibiotic resistance profiles that were similar to the observed for the Shirota and CPB controls. TUCO-16 and TUCO-17 strains were sensitive to amoxicillin, erythromycin, ampicillin, and tetracycline.

**Table 4:** Presumptive safety characteristics of the selected strains isolated from canine milk and their control strains.

**Strains**	**Hemolysis**	**Gelatinase**	**Resistant**
TUCO-16	Negative	Negative	AK, CN, MTZ, VA
TUCO-17	Negative	Negative	AK, CN, MTZ, VA
TUCO-4	Negative	Negative	AK, CIP, CN, MTZ, VA
Shirota	Negative	Negative	AK, CIP, CN, MTZ, VA
CPB	Negative	Negative	AK, CIP, CN, MTZ, VA

AK, amikacin; CIP, ciprofloxacin; CN, gentamicin; MTZ, metronidazole; VA, vancomycin.

### 3.3 Canine LAB strains immunomodulation factors expression in macrophages

**Figure 1:**
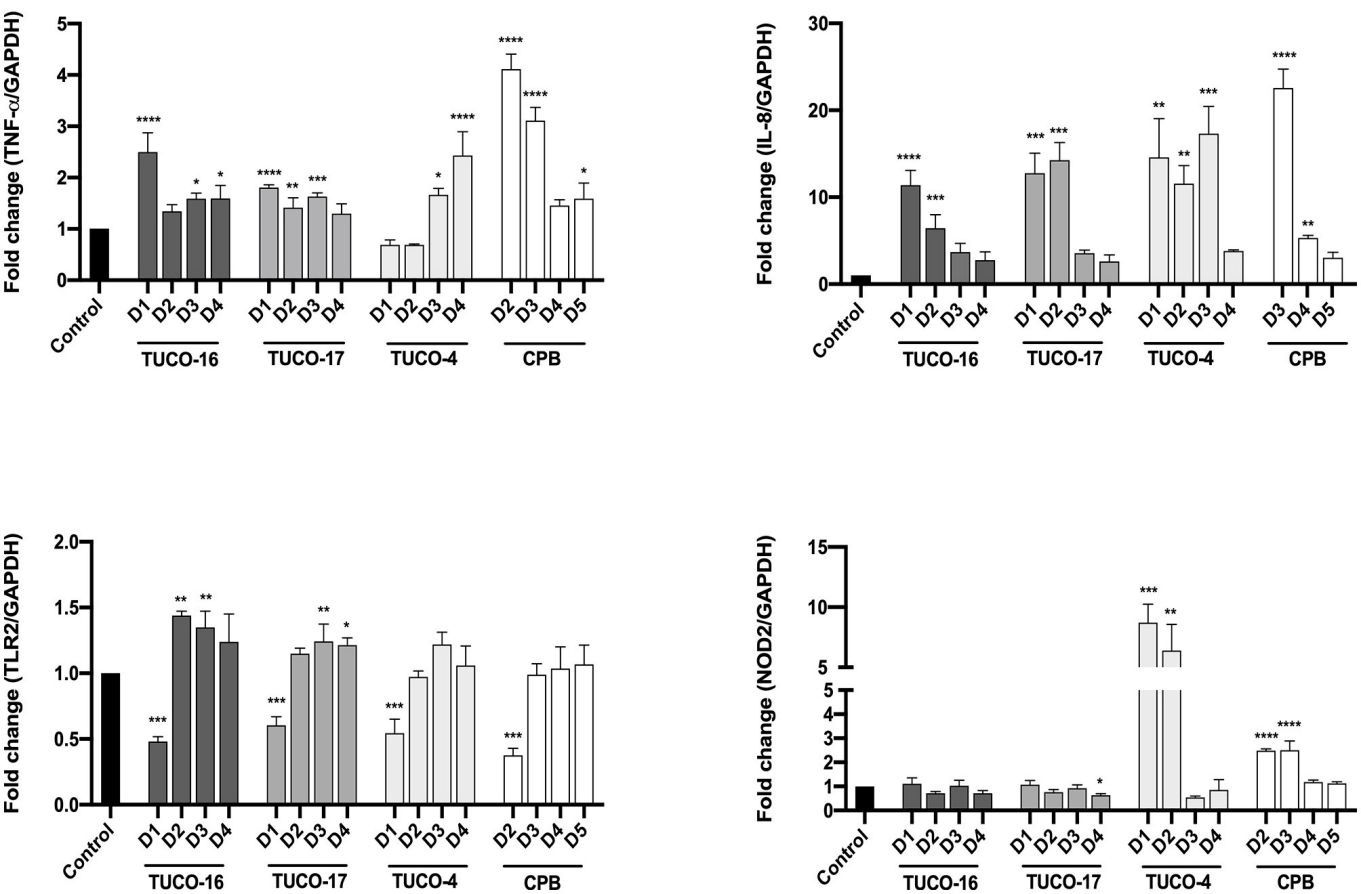
Relative expression of pro-inflammatory cytokines in DH82 cells stimulated with canine lactic acid bacteria. The relative expression of TNF-α, IL-8, TLR2, and NOD2 was determined after 12 h of stimulation. D1: 1 × 10^9^ CFU/mL, D2: 1 × 10^8^ CFU/mL, D3: 1 × 10^7^ CFU/mL, D4: 1 × 10^6^ CFU/mL, D5: 1 × 10^5^ CFU/mL. The probiotic strain TUCO-4 and commercial probiotic BIOPOWER (CPB) were used for comparisons (*p < 0.05; **p < 0.01; ***p < 0.001; ****p < 0.0001).

For the evaluation of the immunomodulatory potential of TUCO-16 and TUCO-17 strains, DH82 canine macrophages were used. A significant increase in the expression of *TNF-*α was observed for the treatments with TUCO-16 and TUCO-17 at 12 h post-stimulation with the highest dose (Figure 1). Similarly, the highest doses of CPB augmented the expression of *TNF-*α. Of note, the two lower doses of TUCO-4 increased the expression of this inflammatory cytokine while no effect was observed with the two higher doses. A clearer dose dependent effect was observed when the expression of *IL-8* was analyzed in canine macrophages after the stimulation with TUCO-16 and TUCO-17 strains (Figure 1). The TUCO-16 and TUCO-17 strains were as effective as the probiotic control TUCO-4 to increase *IL-8*. However, the canine LAB strains were less efficient than the CPB to enhance the inflammatory chemokine.

The expression of the pattern recognition receptors TLR2 and NOD2 were also evaluated in canine macrophages after the stimulation with TUCO-16 and TUCO-17 strains (Figure 1). The highest dose (1 × 10^9^ CFU/mL) of the canine strains significantly reduced the expression of *TLR2*. The same effect was observed for the probiotic controls TUCO-4 and CPB. In contrast, the doses of canine LAB strains inferior to 1 × 10^9^ CFU/mL upregulated the expression of *TLR2*. No significant differences were observed in the expression of *NOD2* when basal levels were compared with those in macrophages stimulated with TUCO-16 and TUCO-17 strains (Figure 1). In contrast, *NOD2* was significantly increased in cells treated with probiotic controls TUCO-4 and CPB.

### 3.4 Canine LAB strains improve resistance to ETEC infection in mice

**Figure 2:**
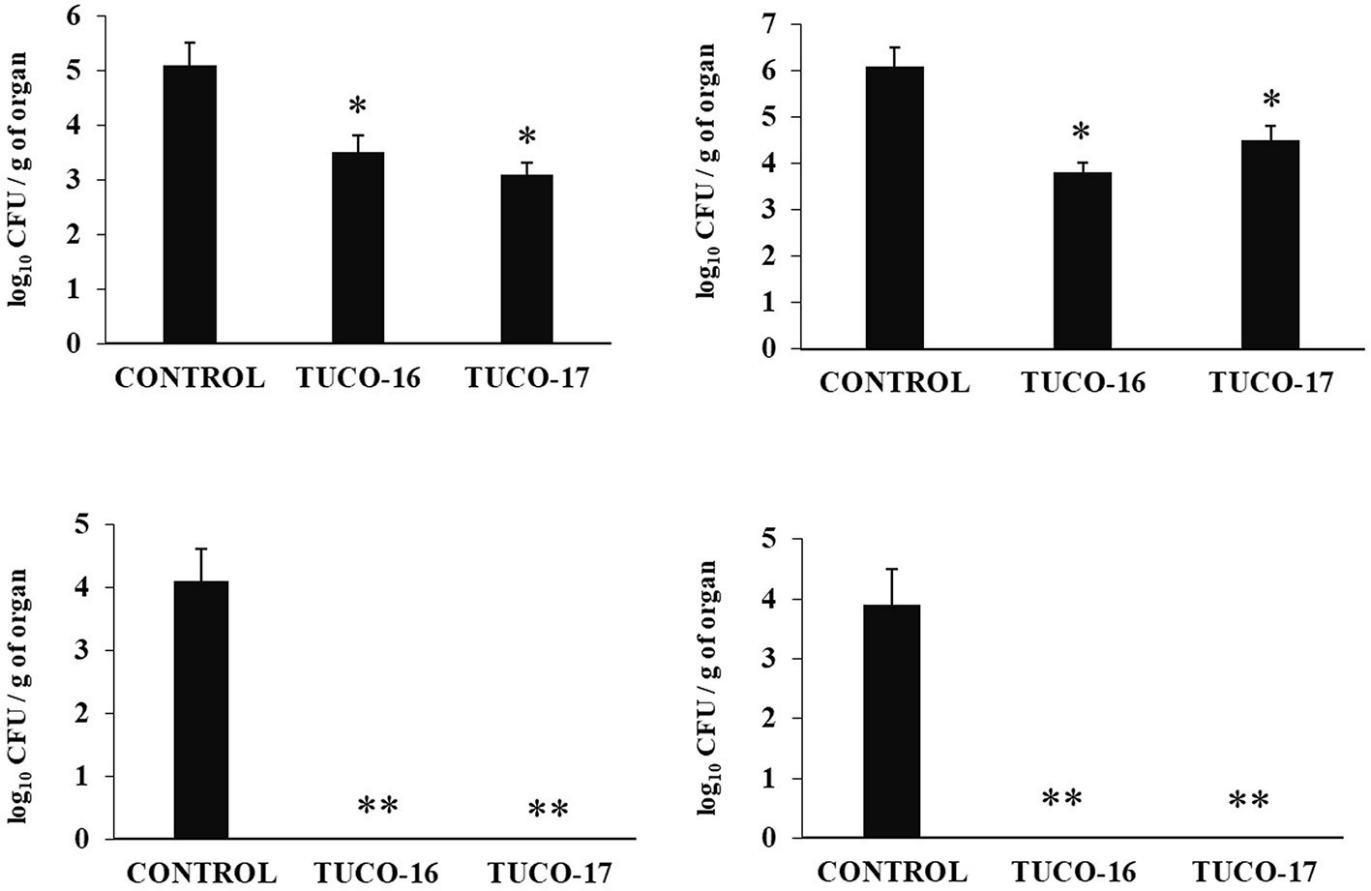
Effect of canine TUCO-16 and TUCO-17 strains on ETEC infection in mice. Mice were orally treated with TUCO-16 or TUCO-17 strains for five consecutive days and then challenged by the oral route with ETEC. Mice with no treatment and challenged with ETEC were used as controls. ETEC counts in jejunum, ileum, spleen, and liver were determined 2 days after the challenge with ETEC. The results represent data from three independent experiments. Values are means ± SD. Asterisks indicate significant differences when compared to the ETEC control group (*p < 0.05, **p < 0.01).

**Figure 3:**
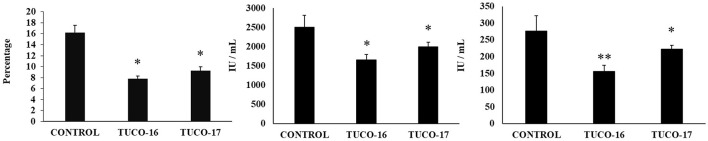
Effect of canine TUCO-16 and TUCO-17 strains on ETEC infection in mice. Mice were orally treated with TUCO-16 or TUCO-17 strains for five consecutive days and then challenged by the oral route with ETEC. Mice with no treatment and challenged with ETEC were used as controls. Body weight loss, serum lactate dehydrogenase (LDH), and serum aspartate aminotransferase (AST) were determined 2 days after the challenge with ETEC. The results represent data from three independent experiments. Values are means ± SD. Asterisks indicate significant differences when compared to the ETEC control group (*p < 0.05, **p < 0.01).

**Figure 4:**
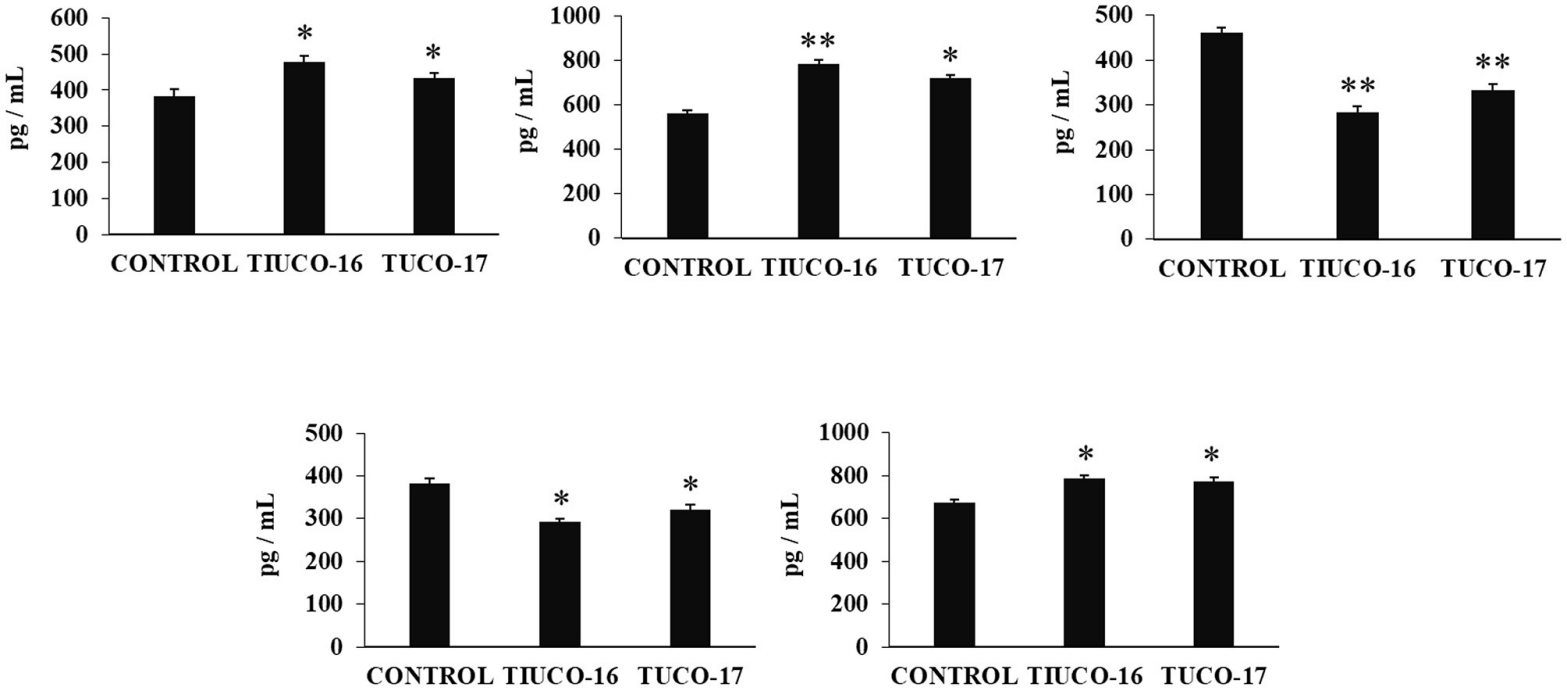
Effect of canine TUCO-16 and TUCO-17 strains on ETEC infection in mice. Mice were orally treated with TUCO-16 or TUCO-17 strains for five consecutive days and then challenged by the oral route with ETEC. Mice with no treatment and challenged with ETEC were used as controls. The intestinal levels of interferon (IFN)-β, IFN-γ, interleukin (IL)-6, IL-10, and tumor necrosis factor (TNF)-α, were determined 2 days after the challenge with ETEC. The results represent data from three independent experiments. Values are means ± SD. Asterisks indicate significant differences when compared to the ETEC control group (*p < 0.05, **p < 0.01).

**Figure 5:**
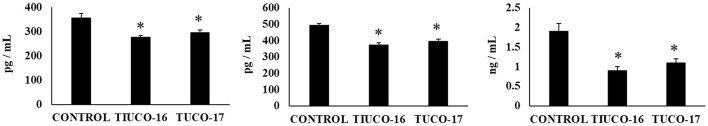
Effect of canine TUCO-16 and TUCO-17 strains on ETEC infection in mice. Mice were orally treated with TUCO-16 or TUCO-17 strains for five consecutive days and then challenged by the oral route with ETEC. Mice with no treatment and challenged with ETEC were used as controls. The intestinal levels of interleukin (IL)-15, chemokine KC (or CXCL1), and monocyte chemoattractant protein 1 (MCP-1) were determined 2 days after the challenge with ETEC. The results represent data from three independent experiments. Values are means ± SD. Asterisks indicate significant differences when compared to the ETEC control group (*p < 0.05).

The ability of the canine TUCO-16 and TUCO-17 strains to improve the resistance against ETEC infection was evaluated. A model of ETEC infection in mice (26) was used for this purpose. The challenge of mice allowed the colonization of ETEC in jejunum, ileum, liver, and spleen as shown by the bacterial cell counts in these tissues (Figure 2). The intestinal pathogen was not detected in blood samples in this time point post-infection. Mice preventively treated with TUCO-16 or TUCO-17 strains had significantly lower ETEC counts in jejunum and ileum. Furthermore, animals that received the canine LAB strain had undetectable levels of the pathogen in liver and spleen (Figure 2). In line with the improved resistance against ETEC challenge, mice treated with TUCO-16 or TUCO-17 strains showed significantly lower loss of body weight and reduced levels of the biochemical markers of injury LDH and AST when compared to controls (Figure 3). As reported previously (26), ETEC infection in mice induced an increase in the levels of proinflammatory cytokines IFN-β, IFN-γ, IL-6, and TNF-α (Figure 4) and chemokines KC, MCP-1, and IL-15 (Figure 5) in the intestine. In addition, an increase in the regulatory cytokine IL-10 was observed in the intestinal fluid of ETEC-challenged animals. Of note, mice preventively treated with TUCO-16 or TUCO-17 strains had significantly higher levels of intestinal IFN-β, IFN-γ, and IL-10 than controls. Canine LAB-treated animals also had lower levels of intestinal IL-6, TNF-α (Figure 4), KC, MCP-1, and IL-15 (Figure 5) than controls.

### 3.5 Canine LAB strains improve resistance to *Salmonella* infection in mice

**Figure 6:**
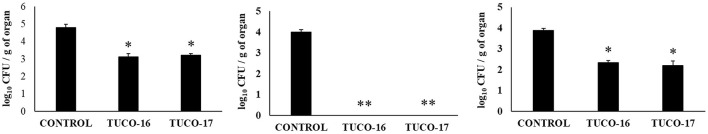
Effect of canine TUCO-16 and TUCO-17 strains on *Salmonella* infection in mice. Mice were orally treated with TUCO-16 or TUCO-17 strains for five consecutive days and then challenged by the oral route with *Salmonella*. Mice with no treatment and challenged with *Salmonella* were used as controls. *Salmonella* counts in blood, spleen, and liver were determined 2 days after the challenge with *Salmonella*. The results represent data from three independent experiments. Values are means ± SD. Asterisks indicate significant differences when compared to the *Salmonella* control group (*p < 0.05, **p < 0.01).

**Figure 7:**
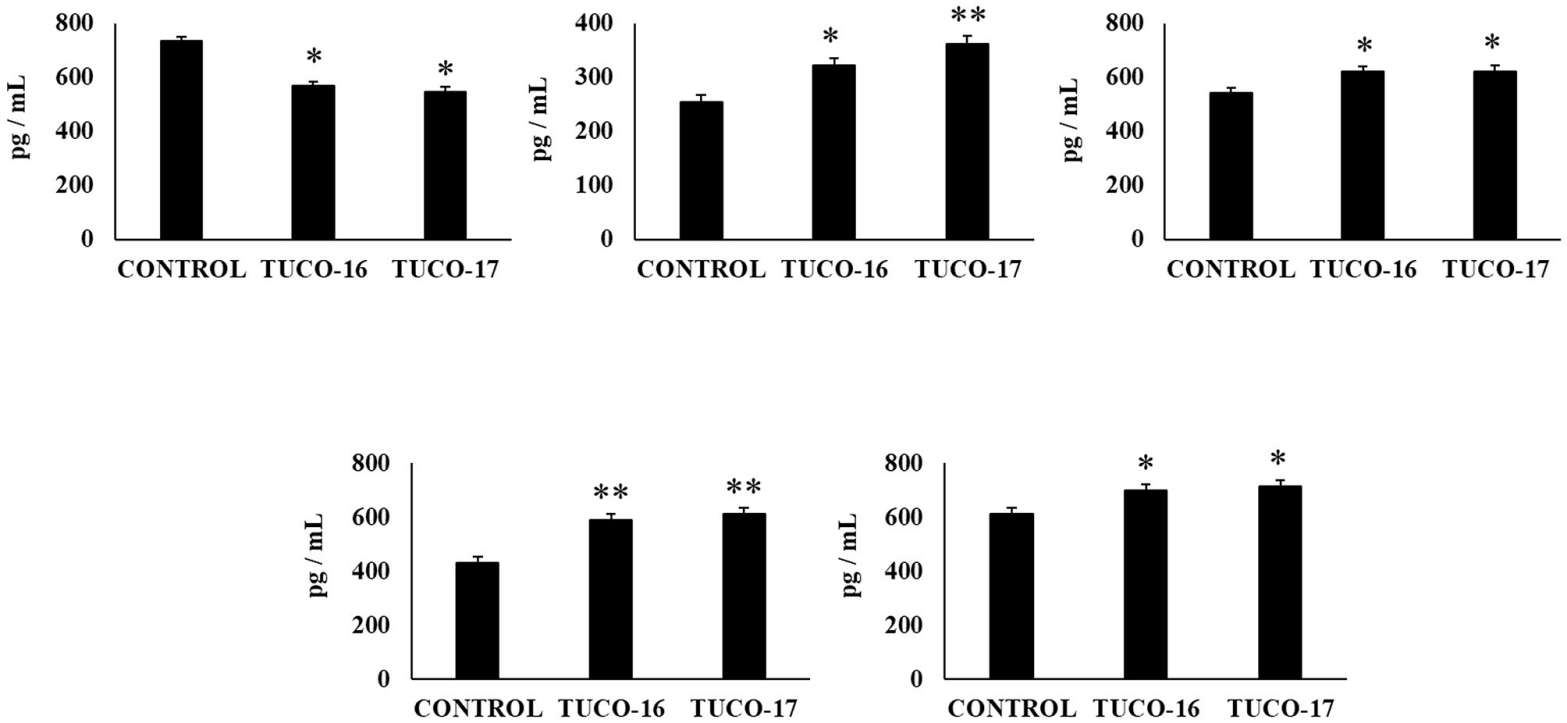
Effect of canine TUCO-16 and TUCO-17 strains on *Salmonella* infection in mice. Mice were orally treated with TUCO-16 or TUCO-17 strains for five consecutive days and then challenged by the oral route with *Salmonella*. Mice with no treatment and challenged with *Salmonella* were used as controls. The intestinal levels of interferon (IFN)-γ, interleukin (IL)-1β, IL-6, IL-10, and tumor necrosis factor (TNF)-α were determined 2 days after the challenge with *Salmonella*. The results represent data from three independent experiments. Values are means ± SD. Asterisks indicate significant differences when compared to the *Salmonella* control group (*p < 0.05, **p < 0.01).

**Figure 8:**
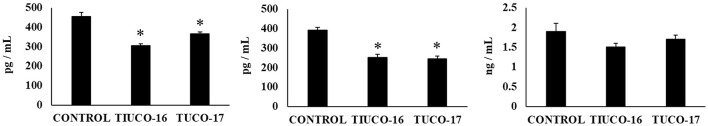
Effect of canine TUCO-16 and TUCO-17 strains on *Salmonella* infection in mice. Mice were orally treated with TUCO-16 or TUCO-17 strains for five consecutive days and then challenged by the oral route with *Salmonella*. Mice with no treatment and challenged with *Salmonella* were used as controls. The intestinal levels of interleukin (IL)-15, chemokine KC (or CXCL1), and monocyte chemoattractant protein 1 (MCP-1) were determined 2 days after the challenge with *Salmonella*. The results represent data from three independent experiments. Values are means ± SD. Asterisks indicate significant differences when compared to the *Salmonella* control group (*p < 0.05).

Finally, the capacity of the canine TUCO-16 and TUCO-17 strains to improve the resistance against *Salmonella* infection was evaluated in a mice model (27). The pathogen was detected in blood, liver, and spleen samples in control mice (Figure 6). Mice preventively treated with TUCO-16 or TUCO-17 strains had significantly lower *Salmonella* counts in liver and spleen. Furthermore, animals that received the canine LAB strain had undetectable levels of the pathogen in blood samples. The challenge with the pathogen augmented the levels of cytokines TNF-α, IL-1β, IL-6, IFN-γ (Figure 7) and the chemokines KC, MCP-1, and IL-15 (Figure 8) in the intestine. Significantly lower levels of the proinflammatory factors TNF-α, KC, and MCP-1 were found in mice preventively treated with the canine TUCO-16 or TUCO-17 strains. In addition, canine LAB stimulated a higher production of IL-1β, IL-6, IFN-γ, and IL-10 in the intestine of Salmonella-challenged animals. Of note, no differences were found in the levels of intestinal IL-15 when control and TUCO-16- and TUCO-17-treated mice were compared (Figure 8).

## 4 Discussion

The administration of probiotics to the diet of companion animals has increased in the last years with the aim of generating beneficial effects on the gastrointestinal health (28). As for humans, canine probiotics should be selected considering the absence of deleterious effects like hemolytic activity or the presence of antibiotic resistance genes, as well as for their benefits including the production of lactic acid and bacteriocins, their adhesion to intestinal epithelial tissues, inhibitory effects on the growth of pathogens, and their immunomodulatory potential (29–31). We aimed to select potential probiotics strains from canine milk and colostrum with the aim of using them in a new canine probiotic formulation with the capacity to prevent gastrointestinal infectious disorders in dogs. Most of the candidate probiotic strains have been isolated from dog's feces while canine milk and colostrum were less explored. Studies evaluating the ability of LAB isolated form dog's milk to resist the gastrointestinal tract conditions, to produce antimicrobial compounds and adherence to intestinal mucin indicate that there are promising strains for future applications as canine probiotics (32). Interestingly, it was reported that the administration of *Lacticaseibacillus rhamnosus* MP01 and *Lactiplantibacillus plantarum* MP02, isolated from canine milk to 1-month-old puppies resulted in a significant preventive effect of gastrointestinal infections (33).

In this work, canine LAB were evaluated according to their abilities to resist to acidic pH, bile salts, and pancreatin concentrations, and the inhibitory effects against gastrointestinal pathogens. Among the evaluated strains, we found that the canine TUCO-16 and TUCO-17 strains are able to induce inhibition halos > 20 mm on Gram negative pathogenic bacteria strains, particularly ETEC and *Salmonella*. This is in line with previous reports describing that isolates from dog feces have shown good antimicrobial activity against multiresistant and foodborne pathogenic bacteria (18). Interestingly, it was shown that there is a relationship between LAB content and the absence of *Salmonella* sp. in dogs' feces (34). It was also demonstrated that *Ligilactobacillus salivarius* strains isolated from feces of *Salmonella*-negative dogs efficiently inhibit the growth of *Salmonella* Typhimurium *in vitro* (19). The milk canine strains *L. rhamnosus* MP01 and *L. plantarum* MP02 also showed inhibition effect against the growth of Gram negative pathogens *in vitro*, including *E. coli* MP07 (O157:H7) (33). In addition, the canine TUCO-16 and TUCO-17 strains were negative for hemolytic and gelatinase activity and had antibiotics resistance profiles similar to the probiotic control strains, which indicates a presumptive innocuousness of the canine bacteria, reinforcing their usefulness as potential probiotics.

Macrophages in the intestinal mucosa can have both pivotal role in the maintenance of homeostasis and contribute to inflammation (35). Reports revealed the predominance of resident macrophages displaying an anti-inflammatory phenotype in the intestinal mucosa of healthy dogs, although they are capable to trigger inflammatory responses against pathogens. Immunohistochemical studies demonstrated that the number of CD163^+^CD204^+^ macrophages are significantly higher in the villus compared to the crypt area, which reflect the higher antigenic load in the small intestinal villus region compared to crypts, including commensal bacteria (35). In addition, the work found that small numbers of CD64^+^ macrophages directly underneath the epithelial layer sending transepithelial projections into the lumen (35). These results indicate, like mice and humans, that canine macrophages are one of the first immune cells that contact orally administered probiotic microorganisms in the gut. Then, the evaluation of the interaction of canine macrophages with LAB strains can be a useful *in vitro* tool to screen and characterize immunomodulatory probiotics. Thus, we also aimed to evaluate whether the canine TUCO-16 and TUCO-17 strains exerted immunomodulatory effects using the DH82 canine macrophage cell line. We demonstrated that both strains could induce an upregulation of *TNF-*α and *IL-8* as well as the PRRs *NOD-2* and *TLR2*, which are immune factors differentially regulated in macrophages by probiotic strains (36, 37). It was reported that probiotic lactobacilli are able to induce the production of proinflammatory cytokines such as IL-8, TNF-α, IL-12p70, and IL-6 (36) and TLR2 (37) in macrophages, which in turn activate their phagocytosis and bactericidal activity, and improve their interactions with other immune cell populations of the gut.

The results obtained *in vitro* with the canine TUCO-16 and TUCO-17 strains suggest that both strains would be capable of differentially modulating intestinal immunity. To demonstrate this effect *in vivo*, two mouse models of infection were used: ETEC and *Salmonella* infection, considering that both Gram negative pathogens can infect dogs. *Salmonella* is frequently isolated from both healthy and diarrheic dogs at the same prevalence (38). The prevalence of this bacterium has been shown to be much higher in dogs that are fed raw food diets. For example, *Salmonella* was isolated from 80% of the diet samples and 30% of the stool samples of dogs fed raw chicken diets (39). In addition, contaminated foods, including unprocessed or raw dog food, especially raw meat, has been related as one of the most important risk factors of *Salmonella* carriage in dogs (40). The infection in dogs is in many cases subclinical, but it can induce symptoms including malaise, anorexia, vomiting, abdominal pain, and diarrhea (38). Furthermore, some dogs may manifest clinical signs of sepsis. On the other hand, *E. coli* is part of the normal intestinal microbiota of dogs (38), but can be associated with gastroenteritis in the presence of bacterial virulence factors and impaired immunity. In this regard, it was shown that ETEC in the environment enter dogs via the oral route, transit and colonize the small intestine where they can multiply rapidly (41). It is assumed that the degree of colonization determines whether or not disease will result from infection. Once established, ETEC can synthesize and secrete one or more types of enterotoxins, which induce the secretion of water and electrolytes in the intestinal lumen. Then, ETEC can cause a rapid onset of secretory diarrhea leading to severe dehydration and electrolyte imbalance.

In our hands, mice preventively treated with canine TUCO-16 or TUCO-17 strains had an improved resistance to ETEC and *Salmonella* challenges, as demonstrated by the significantly lower pathogen counts in the infected tissues. As expected, these beneficial effects were related to a differential modulation of the intestinal immune response. A distinct intestinal cytokine profile was found in mice treated with canine LAB, which was characterized by higher levels of IFN-γ and IL-10, and lower concentrations of TNF-α, KC and MCP-1 than controls, in both infection models. Several studies have reported that the most remarkable effect of human probiotic strains on intestinal cytokine dynamics is the increase of IFN-γ, and the regulatory cytokine IL-10 (27, 42). In this regard, it was shown that the administration of the immunostimulatory probiotic strains to mice can enhance the activation of intestinal and peritoneal macrophages as well as Peyer's patches CD4^+^IFN-γ^+^ T cells (27, 42–44). These results allow us to speculate that orally administered canine TUCO-16 or TUCO-17 strains could exert an immunomodulatory effect on macrophages acting directly on them or indirectly through the cytokines produced by other immune cells like T cells. These improved functions of intestinal immune cells would account for the enhanced resistance against bacterial pathogens.

Activation of immune cells and cytokine production are of importance in the defense of the intestinal mucosa against pathogens like ETEC and *Salmonella*. Although this mechanism represents an important primary line of host defense, prolonged or dysregulated proinflammatory cytokine production may lead to tissue damage and epithelial barrier dysfunction (14). Thus, proper regulation of the inflammatory response is necessary for complete and efficient protection against intestinal pathogens. The enhanced levels of IL-10 and lower concentrations of TNF-α, KC, and MCP-1 induced by the treatments with canine TUCO-16 or TUCO-17 strains indicate that mice were also protected against the inflammatory damage. This is in line with studies demonstrating that probiotics can mitigate damaging immune responses during Gram negative bacterial infections (45, 46).

## 5 Conclusions

The *in vitro* and *in vivo* studies conducted in this work demonstrate the probiotic potential of strains TUCO-16 and TUCO-17, which were isolated from canine colostrum and milk. These strains have shown resistance to simulated gastrointestinal conditions and inhibitory effects against recurrent pathogens in gastrointestinal infections, as well as immunomodulatory properties. Typically, probiotic products for companion animals, such as dogs, are not extensively studied to validate the criteria necessary to be considered probiotics. However, we have convincingly demonstrated the probiotic potential of canine milk-derived strains for future applications in the treatment and prevention of gastrointestinal infections in dogs.

In the immediate future, it is necessary to conduct trials to validate the beneficial effects directly in the canine host. Additionally, complementary analyses are needed to explain the cellular and molecular modes of action of the TUCO-16 and TUCO-17 strains to supplement the characteristics of the probiotics described here.

## Data availability statement

The raw data supporting the conclusions of this article will be made available by the authors, without undue reservation.

## Ethics statement

The animal study was approved by Comité de Ética, Bioética y Bioseguridad de la Vicerrectoría de Investigación y Desarrollo de la Universidad de Concepción. The study was conducted in accordance with the local legislation and institutional requirements.

## Author contributions

SQ-V: Conceptualization, Formal analysis, Supervision, Writing—review & editing, Data curation, Investigation, Methodology, Writing—original draft. CM-F: Conceptualization, Formal analysis, Investigation, Methodology, Writing—original draft, Writing—review & editing, Funding acquisition. AP: Formal analysis, Investigation, Writing—original draft, Data curation, Methodology. PB: Formal analysis, Investigation, Writing—original draft. FS: Investigation, Methodology, Writing—review & editing, Data curation, Supervision. AA: Data curation, Investigation, Methodology, Formal analysis, Writing—original draft. BP: Formal analysis, Investigation, Methodology, Writing—review & editing. NP: Data curation, Formal analysis, Investigation, Methodology, Visualization, Writing—review & editing. CA: Conceptualization, Methodology, Formal analysis, Writing—review & editing, Funding acquisition. LA: Conceptualization, Formal analysis, Methodology, Data curation, Investigation, Writing—original draft. JV: Conceptualization, Data curation, Investigation, Methodology, Writing—original draft, Visualization, Writing—review & editing. JT: Conceptualization, Writing—review & editing, Formal analysis, Funding acquisition, Project administration, Supervision, Validation, Visualization.
