# Supplementary figures and images for: Isolation, characterization, and immunomodulatory activity evaluation of probiotic strains from colostrum and canine milk
Source: Front Vet Sci. 2023 Nov 23;10:1266064. doi: 10.3389/fvets.2023.1266064 (PMC10701529; doi:10.3389/fvets.2023.1266064)

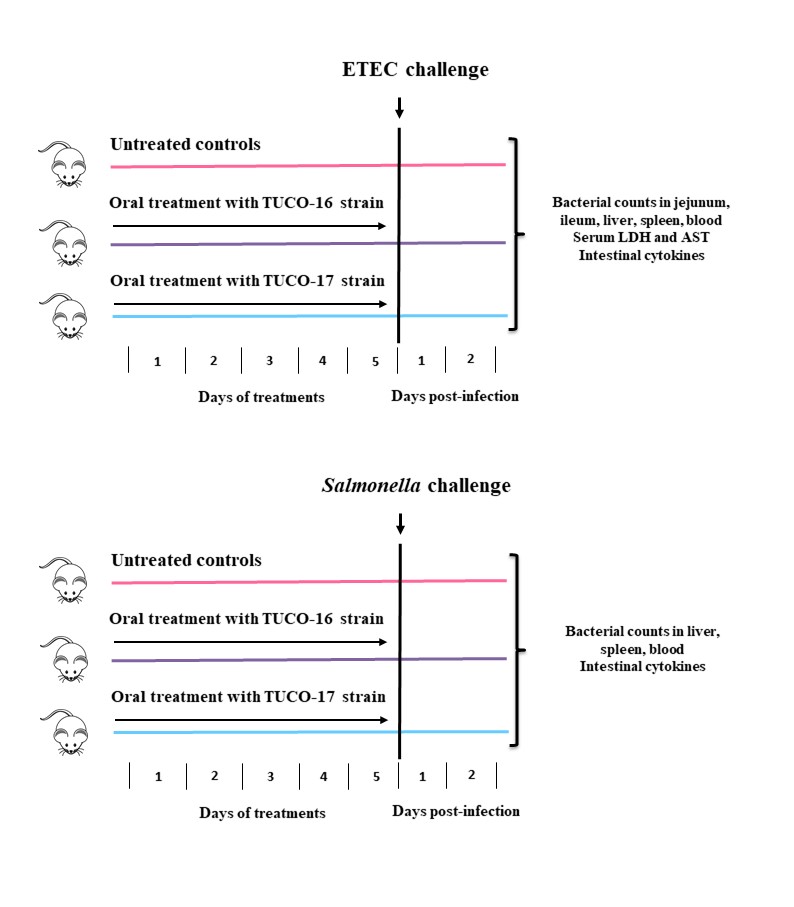

Supplement: Supplementary file 1 [file Image_1.JPEG]
